# Tapering pressure of wall vacuum-assisted closure for the treatment of patients with pancreatic fistula in traumatic pancreatic injury: Report of two cases

**DOI:** 10.1016/j.ijscr.2020.02.048

**Published:** 2020-02-28

**Authors:** Adeodatus Yuda Handaya, Aditya Rifqi Fauzi, Victor Agastya Pramudya Werdana

**Affiliations:** Digestive Surgery Division, Department of Surgery, Faculty of Medicine, Universitas Gadjah Mada/Dr. Sardjito Hospital, Yogyakarta 55281, Indonesia

**Keywords:** Pancreatic fistula, Pancreatic trauma, Tapering pressure wall suction, NPWT, VAC

## Abstract

**Introduction:**

Traumatic injury to the pancreas is rare and difficult to diagnose, requiring immediate operative management. It also has high mortality and morbidity rates. Postoperative pancreatic fistula is one of the complications that is considered a nightmare for digestive surgeons. The prevalence of POPF is estimated at 13%–41%, with 28% of mortality rate and the most common cause of death is retroperitoneal sepsis and hemorrhage. It requires complex treatment and a long duration of hospitalization of patients with a large cost burden.

**Presentation of case:**

Here we report 2 cases of POPF after pancreatic injury in abdominal trauma. The patients underwent emergency laparotomy. In the hospital ward, the patients developed wound dehiscence and a clear viscous pancreatic juice came out from the wound with high output. The installation of wall VAC using wall suction with pressure adjustments according to the number of products per day was performed. The patients showed good outcomes, the pancreatic juice output decreased and diminished, and the wound also narrowed and closed.

**Discussion:**

VAC using wall suction is a device that applies the technique of NPWT and an emerging procedure used to treat patients with complex wounds. NPWT can reduce pooling of fluid, while reducing shear stress and tissue hypoxia at the wound edges, and stimulating the release of vascular endothelial growth factor in wound milieu.

**Conclusions:**

Tapering pressure of VAC using wall suction for treatment of pancreatic fistula in post laparotomy pancreatic injury patients is a simple and easy procedure with good outcomes.

## Highlights

• Postoperative pancreatic fistula is a nightmare for digestive surgeons.
• Requires complex treatment and a long duration of hospitalization of patients with a large cost burden.
• VAC using wall suction with NPWT principle can be used to treat patients with complex wounds.
• A simple and easy procedure with good outcomes.

## 1 Introduction

The pancreas is an organ that is rarely injured in trauma, but is associated with high mortality and morbidity rates. The mortality rate is between 9%–34%. Injury to the pancreas caused by penetrating trauma is around 20%–30% which requires immediate operative management [1].

One of the postoperative complications due to trauma to the pancreas is postoperative pancreatic fistula (POPF). POPF is an abnormal communication between the ductal pancreatic epithelium and other epithelial surfaces that contain pancreatic-derived, enzyme-rich fluid. This condition is related to pancreatic-enteric anastomosis leakage, but can also involve injury to the pancreas. Until now, POPF is still considered the most dangerous complication after pancreatic resection [2]. The POPF definition agreed by consensus is the drain output of any measurable volume of fluid on or after postoperatively day 3 with an amylase content greater than 3 times the serum amylase activity. POPF is also divided into three grades: patients with relatively benign clinical courses (Grade A), moderately unwell patients requiring medical or minimally invasive intervention (Grade B), and critically ill patients, often with sepsis, requiring invasive intervention (Grade C) [3].

POPF is the main source of patient morbidity and mortality after pancreatic resection, and the prevalence is estimated at 13%–41%. POPF is a serious concern because it requires complex treatment and a long duration of hospitalization of patients with a large cost burden [3]. The mortality rate of patients with major pancreatic fistula is up to 28% and the most common causes of death are retroperitoneal sepsis and hemorrhage [3]. In cases of pancreatic injury, peritonitis is also usually found after sharp or blunt trauma intraoperatively. During surgery, identifying injuries to the pancreatic duct is very difficult. Operations management includes debridement and peripancreatic drain installation.

Vacuum Assisted Closure (VAC) is one of the wound therapies to promote open wound healing. VAC application is based on the negative pressure wound therapy (NPWT) technique [5,6]. VAC therapy promotes healing of the wound by helping the promotion of granulation tissue and wound edge approximation. This therapy has been widely studied and proven effective in helping wound healing, leading not only to economic advantages, but especially to a noticeably improved patient health [6].

The aim of this report was to discuss the usage of VAC with tapering pressure using wall suction in management of POPF, with a focus on potential future directions for research in this field. In this report we describe a modified method of pressure adjustment therapy on wall suction connected VAC, where the pressure is set at −150 mmHg for products more than 500cc/24 h, −100 mmHg for products 250–500cc/24 h, −50 mmHg if the product is less than 250 cc, and if the product is less than 50cc/24 h the patient can be treated as outpatient. This report is a perspective study from the first author as the digestive surgeon who operated on the patients. This research work has been reported in line with the SCARE checklist [7].

## 2 Presentation of case

### 2.1 Case 1

**Fig. 1:**
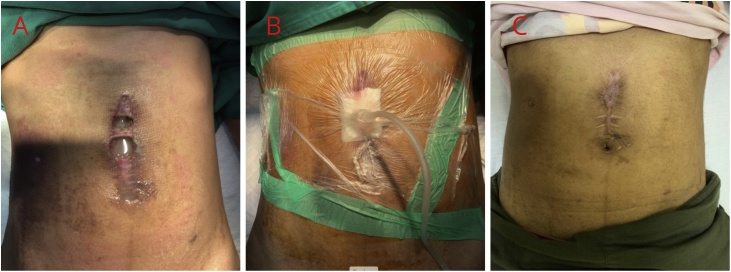
A) Pancreatic fistula 2 weeks after laparotomy. B) Vacuum assisted closure using wall connected vacuum. C) 3 months postoperative.

A 32-year-old woman presented to the emergency room because of a traffic accident, in which the patient’s abdomen hit the steering. We performed an exploratory laparotomy. At the time of surgery, a grade 2 laceration was found in the pancreas, and an installation of peripancreatic drainage was performed. One week postoperatively, drainage was ineffective and right quadrant peritonitis occurred. There was also a wound dehiscence in the supraumbilical area and a clear viscous pancreatic juice came out with an output of 300–500 cc per day (Fig. 1a). The patient was then placed on abdominal wall suction (Fig. 1b) with pressure adjustments according to the number of products per day: −100 mmHg for products 250–500 cc, −50 mmHg if less than 250cc, and if less than 50 cc patients can be treated as an outpatient. After 4 weeks of open treatment after VAC treatment, the wound was shrinking in size, and the fistula and the wound closed in the third month after trauma (Fig. 1c).

### 2.2 Case 2

**Fig. 2:**
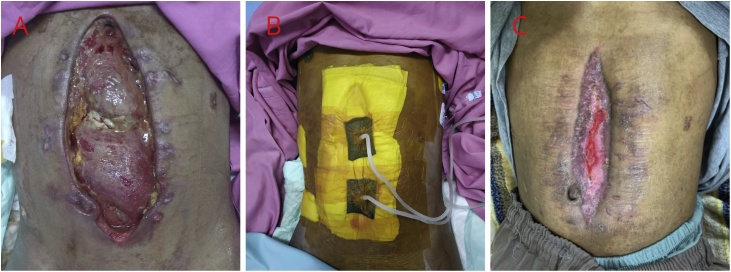
A) Burst abdomen due to a complicated abdominal infection caused by pancreatic fistula. B) VAC connected to wall vacuum installation in the patient’s abdomen. C) 3 months postoperative.

A 20-year-old man suffered a stab wound in the upper left abdomen. He was hemodynamically unstable, with blood pressure 70 per palpation. Accordingly, the patient underwent resuscitation followed by emergency laparotomy. Intraoperatively, we found liver lacerations of segment 3 AAST grade 2, and performed hepatorraphy. We also found lacerations in the grade 2 AAST in pancreatic corpus parenchyma, and while performing primary suturing, a non-pulsating extensive hematoma in the retroperitoneal was also found. After installing peripancreatic and pelvic region drainage, on the 7th day after surgery, the patient had peritonitis and wound dehiscence and fistula in the epigastrium. A serohemorrhagic viscous liquid product also came out with fluid from the pancreas. There were also complications of sepsis and intrabdominal compartment syndrome. Following debridement, re-laparotomy, pancreatic duct ligation, and open abdominal wound management (Fig. 2a), installation of an abdominal vacuum was connected to the wall suction machine (Fig. 2b). The initial product of the patient after re-surgery and debridement was 500–750 cc per day, so our patient was then placed on the abdominal wall suction with pressure adjustments according to the number of products per day, where the pressure was −150 mmHg for products more than 500cc, −100 mmHg for products 250–500cc, −50 mmHg if less than 250cc, and if less than 50cc patients can be treated openly. After 4 weeks of open treatment after VAC treatment, the wound narrowed, and the fistula and the wound closed in the third month after trauma (Fig. 2c).

## 3 Surgical procedures

**Fig. 3:**
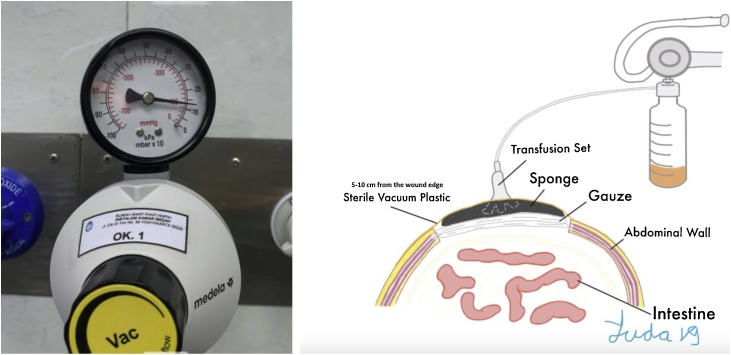
A) Schematic diagram of VAC installation in the patient’s abdomen. B) Pressure regulator connected to wall vacuum.

1. We opened the abdomen for dehiscence, then did debridement and necrotomy.
2. Next, the wound was covered with wet sterile gauze
3. Then we used a sterile foam-based sponge (pore sizes from 400 to 600 μm) which was connected to a transfusion set.
4. After that we wrapped it with sterile draping along 5–10 cm from the lateral margin of the wound.
5. Then, it was connected to the wall suction with tapering pressure depending on the volume of patient’s fistula products.
6. Sponges and dressings were replaced every 3 days. The illustrative installation and tool drawings can be seen in Fig. 3.

## 4 Discussion

The significance of POPF is in the fact that this condition is a life-threatening complication, and it prolongs the hospital stay and adds to hospital costs [4,8]. One risk factor for POPF is high pancreatic juice output, which is defined if the output is more than 200cc in 24 h [4], as happened in our cases. Another factor that contributes to the patient's condition is infection. Infection places a heavy burden on costs and increases the risk of intra-hospital mortality. With an average cost of US $11,000 per complication of infection wounds after pancreatectomy surgery and an additional 6.5 days hospital stay, this represents a significant burden on the health system [9]. To overcome this slow and costly recovery, the wall suction using the NPWT technique is used to treat patients in our hospital. NPWT can reduce pooling of fluid, which can facilitate bacterial growth, as well as reduce shear stress and tissue hypoxia at the wound edges, and stimulate the release of vascular endothelial growth factor in wound milieu [9]. In addition, the film dressing is also waterproof so it prevents pathogens from the skin entering the wound [6].

It has also been proven that treating wounds with subatmospheric pressure of −125 mmHg can increase tissue perfusion fourfold at the edge of the tissue. The use of VAC in other types of wounds such as diabetic foot wounds also gives good results, as described in Lavery et al. [10] who examined the effectiveness of using NPWT with low pressure (80 mmHg). Umezawa et al. [11] also reported good wound healing results in two patients with head and neck, and esophageal cancer who underwent resection and reconstructive surgery. A similar study by Prichayudh et al. using a VAC wall found that this technique was very effective in containing a large amount of fistula products from multiple fistula openings. Also, it is relatively inexpensive, costing around $60 USD per set. Decrease in bacterial load was also found in previous study in wounds treated with NPWT [12].

The optimal pressure to be applied for improvement of the POPF wound is not currently known. In general, the NPWT pressure used to achieve good healing response is from −75 mmHg to −150 mmHg [10,13]. Additionally, there are several pressure management techniques in NPWT, namely: continuous mode, intermittent pressure therapy, and variable pressure therapy. In continuous mode, the pressure used is −80 mmHg constant. In the intermittent pressure therapy, the pressure is changed between 0 and −80 mm Hg repeatedly. Whereas in the variable pressure technique, the pressure is changed from −10 to −80 slowly. All of these techniques show equal results in the process of wound healing, while considering the use of NPWT should be adjusted to the patient’s condition [14,15]. Other studies have shown that the use of a low pressure NPWT of −80 mmHg and using sterile gauze produce good wound healing. Additionally, the use of low pressure NPWT is considered effective to be applied to circumferential injuries [10].

Finally, although our methods showed good outcomes, the current available evidence does not allow us to conclude that this procedure is effective for wider characteristics of patients with POPF. Further study should asses in detail the safety and effectiveness of the procedure and the tapering pressure classification of VAC.

## 5 Conclusions

The present study demonstrates that wall suction tapering pressure VAC therapy is a safe and reliable option in traumatic pancreatic fistula with high output. This technique can be used as an alternative procedure to treat this type of patient. Further prospective studies with larger samples and longer follow-up period are needed to confirm and clarify our findings.

## Sources of funding

The authors declare that this study had no funding resource.

## Ethical approval

The informed consent form was declared that patient data or samples will be used for educational or research purposes. Our institutional review board also do not provide an ethical approval in the form of case report.

## Consent

Written informed consent was obtained from the patient for publication of this case report and accompanying images. A copy of the written consent is available for review by the Editor-in-Chief of this journal on request

## Author contribution

Adeodatus Yuda Handaya conceived the study and approved the final draft. Aditya Rifqi Fauzi, Victor Agastya Pramudya Werdana drafted the manuscript and critically revised the manuscript for important intellectual content. Adeodatus Yuda Handaya, Aditya Rifqi Fauzi, and Victor Agastya Pramudya Werdana facilitated all project-related tasks.

## Registration of research studies

This is not a ‘first in humans’ report, so it is not in need of registration.

## Guarantor

Adeodatus Yuda Handaya.

## Provenance and peer review

Not commissioned, externally peer-reviewed.

## CRediT authorship contribution statement

**Adeodatus Yuda Handaya:** Conceptualization, Methodology, Resources, Writing - original draft. **Aditya Rifqi Fauzi:** Writing - review & editing, Resources, Validation. **Victor Agastya Pramudya Werdana:** Writing - review & editing, Resources, Validation.

## Declaration of Competing Interest

No potential conflict of interest relevant to this article was reported.
